# The Fourth Edition of Samuel Gross’ Practical Treatise on Impotency, Sterility and Allied Disorders of the Male Sexual Organs

**Published:** 1891-02

**Authors:** 


The Fourth Edition of Samuel Gross' PRACTICAL TREATISE ON IMPOTENCY, STERILITY AND ALLIED DISORDERS OF THE MALE SEXUAL ORGANS. Revised by F. R. Sturgis, M. D., and published by Lea Bros. & Co., Philadelphia.

As now given to the public, is complete, terse, and instructive.

Although there is little that can be called original, or new, yet, the manner in which it is presented, is interesting, and as a digest, is comprehensive. H. H.

